# Genome skimming identifies polymorphism in tern populations and species

**DOI:** 10.1186/1756-0500-5-94

**Published:** 2012-02-14

**Authors:** David George Jackson, Steven D Emslie, Marcel van Tuinen

**Affiliations:** 1 Department of Biology and Marine Biology, University of North Carolina Wilmington, Wilmington, NC 28403, USA

## Abstract

**Background:**

Terns (Charadriiformes: Sterninae) are a lineage of cosmopolitan shorebirds with a disputed evolutionary history that comprises several species of conservation concern. As a non-model system in genetics, previous study has left most of the nuclear genome unexplored, and population-level studies are limited to only 15% of the world's species of terns and noddies. Screening of polymorphic nuclear sequence markers is needed to enhance genetic resolution because of supposed low mitochondrial mutation rate, documentation of nuclear insertion of hypervariable mitochondrial regions, and limited success of microsatellite enrichment in terns. Here, we investigated the phylogenetic and population genetic utility for terns and relatives of a variety of nuclear markers previously developed for other birds and spanning the nuclear genome. Markers displaying a variety of mutation rates from both the nuclear and mitochondrial genome were tested and prioritized according to optimal cross-species amplification and extent of genetic polymorphism between (1) the main tern clades and (2) individual Royal Terns (*Thalasseus maxima*) breeding on the US East Coast.

**Results:**

Results from this genome skimming effort yielded four new nuclear sequence-based markers for tern phylogenetics and 11 intra-specific polymorphic markers. Further, comparison between the two genomes indicated a phylogenetic conflict at the base of terns, involving the inclusion (mitochondrial) or exclusion (nuclear) of the Angel Tern (*Gygis alba*). Although limited mitochondrial variation was confirmed, both nuclear markers and a short tandem repeat in the mitochondrial control region indicated the presence of considerable genetic variation in Royal Terns at a regional scale.

**Conclusions:**

These data document the value of intronic markers to the study of terns and allies. We expect that these and additional markers attained through next-generation sequencing methods will accurately map the genetic origin and species history of this group of birds.

## Background

To investigate the role of physical boundaries to evolutionary divergence and ecological isolation, specific genomic markers are often targeted. The choice of genomic marker is frequently guided by published and previously tested loci, but also dependent on the mutation rate appropriate for the temporal depth of the study and the quality of the sampled DNA. Mitochondrial and nuclear microsatellite markers are most often used in population studies. For phylogeographic investigations, nuclear sequence-based markers (exonic, intronic or anonymous loci) are becoming increasingly popular [1] especially with access to reference genomes and next-generation sequencing that allows sampling many loci [2]. However, these markers are not widely used yet in population studies due to apparent low level of divergence or lack of access [3]. Thus, for many biologists that study non-model species with a minimal budget for DNA analysis, mitochondrial or microsatellite loci are still the preferred choice. When such researchers work with lineages for which published microsatellite loci have not been developed, the single choice is to focus on mitochondrial markers, where number of appropriate loci, and statistical power, is limited.

A new avenue was recently provided by publication of a large number of autosomal, intronic (exon-priming and intron crossing) markers that were shown to amplify across Neognathous birds [4], many of which also displayed extensive intraspecific variation in a representative songbird. The applicability of these markers in population studies of non-model avian species is promising [1,2,5,6], but remains untested for the majority of birds. Despite the apparent universal nature of these primers, amplification success varied across avian taxa (93% in *Gallus*; 79-85% in Passeridan songbirds; 78% in *Falco*; 34% in *Aegolius*; 26% in *Aquila*; [4,7]. Secondly, because bird orders may differ in genomic mutation rate [8-10] and species vary in effective population size, polymorphic loci in one species may not be useful for other species.

One lineage of non-model birds for which novel genetic markers are especially needed is the subfamily Sterninae, family Laridae, consisting of terns and noddies. Terns are a lineage of cosmopolitan shorebirds with an unresolved evolutionary history that comprises several species of conservation concern. Previous study has left most of the nuclear genome unexplored, and population-level studies are limited to only 15% of the world's terns. The value of mitochondrial DNA for population genetic study is limited in this lineage due to slow rate of evolution in the mitochondrial control region and presence of nuclear copies [11,12]. Also, low cloning efficiency and low overall number make microsatellites an unfavorable choice [12,13]. Hence, polymorphic nuclear sequence loci provide an attractive option to overcome the current challenge in tern population genetics. Such loci would further help gain more insight into the yet unresolved phylogenetic relationship of Sterninae relative to Laridae (gulls) and Rhynchopinae (skimmers), and the linear sequence of tern genera within Sterninae.

### Challenges in tern taxonomy and population genetics

Terns are distinct shorebirds that share many similarities with gulls and skimmers [14,15]. Currently placed in a subfamily (Sterninae: [16]) in the gull family Laridae, terns previously have been given tribe status (Sternini: [17]) or were placed in their own family (Sternidae; [18]) next to Laridae and skimmers (Rynchopidae). The precise phylogenetic relationships between terns, gulls and skimmers remain contested because of apparent conflict between different molecular data sets [19]. On the one hand, mitochondrial (ND2, cytb, 12S rRNA: [14], DNA-DNA hybridization [17], electrophoretic [20] and supertree data [21] agree with the traditional sister group relationship between terns and gulls [[22,23], but see [24]] and place skimmers as sister to this larger group. However, nuclear data alone or in combination with mitochondrial data provide varying results. A combination of RAG1 exon 1 and Myoglobin intron 2 weakly supported the gull-tern grouping, although this grouping was driven primarily by the myoglobin locus [25]. Instead, Bayesian analysis of RAG1 exon 1 alone yielded a grouping of gull+skimmer [26], a result also found with Bayesian analysis of three mitochondrial genes (CO1, cytb, and ND2: [27]) and with RAG1 combined with mitochondrial genes (cytb, ND2, 12S: [28]). Because the latter analysis yielded full Bayesian posterior support for this result, this node was recently used in the timetree of shorebirds [29]. However, a fourth molecular study on shorebirds [30] based on both mitochondrial rRNA and three different nuclear loci (beta-fibrinogen intron 7, GAPDH intron 3-5, and ADH intron 5) yielded a third possible topology, a grouping of terns and skimmers, which had full support for MP analyses and near-significant support from ML analyses (87%). Considering the different rates of mutation and lineage sorting among loci, analyses and taxon sampling regimes, the nature of phylogenetic conflict remains unclear. The source of the conflict may further lie in the short time span during which initial evolutionary diversification took place.

Traditionally, most terns are placed into the genus *Sterna *with few exceptions such as the distinct noddies (*Anous*; [14]). Based on mitochondrial DNA sequence analysis, *Sterna *was split into separate genera of closely related species that bear similar morphological features, particularly feather coloring and size [14]. These new genera are: *Thalasseus *("crested terns"), *Onychoprion *("brown-winged terns"), *Sternula *("little terns"), *Hydroprogne *(Caspian Tern), and *Gelochelidon *(Gull-Billed Tern). A reduced *Sterna *("typical black-capped terns") is maintained along with other traditionally non-*Sterna *terns in the genera *Larosterna *(Inca Tern), *Chlidonias *("marsh terns"), *Phaetusa *(Large-Billed Tern), *Gygis *(Angel Tern) and *Anous *(noddies) [14]. Based on mitochondrial data, noddies continue to be the most basal of the terns, but *G*ygis *alba *is placed in an intermediate position to the noddies and all other terns, and *Onychoprion *is the first offshoot among the other terns [14,27]. Nuclear data continue to support the generic divisions but with a different topology at the base of terns. Nuclear data (alone or in combination with mt data) suggest placement of *Gygis *(and noddies) outside of a grouping of terns, skimmers and gulls, with *Sternula *or *Sternula*+*Phaetusa *as the first branch in Sternidae and *Onychoprion *merged with *Sterna *[27,28].

In addition, tern population genetic studies, although limited in taxonomic scope, has indicated the need for change in tern taxonomy. For instance, recent work [31] using three mitochondrial genes and two nuclear genes in Sandwich Terns (*Thalasseus sandvicensis*) strongly supported the separation into two species that surprisingly are not even each other's closest relatives. One species is comprised of *T*. *s*. *acuflavidus *and *T*. *s*. *eurygnatha *(Cayenne and Cabot's Terns, both New World terns) and *T*. *s*. *sandvicensis *(Sandwich Tern, an Old World Tern) is a different species. In contrast, mitochondrial divisions in the genus *Gygis *do not merit separation of the Little White Tern and White Tern into two species [32]. Despite strong natal philopatry and breeding site fidelity in many species, low genetic differentiation is reported in all tern genetic studies across subspecies and geographic range [13,31-38] suggesting long-term isolation and morphological differentiation due to selection, while experiencing ongoing gene flow (Additional file 1: Table S1).

### Royal tern ecology and conservation

The Royal Tern has a widespread distribution along the Atlantic and Pacific coasts of North America, Central America, and South America, along with being present in the Caribbean and on the West African Atlantic Coast [39]. The Royal Tern consists of two distinct subspecies [18]: the New World Royal Terns (*Thalasseus m. maximus*), and the Old World (West African) Royal Terns (*T. m. albididorsalis*). Population genetic studies have not been undertaken on Royal terns, thus little is known of the degree of gene flow among populations [39]. Banding data in southern North Carolina [39,40] indicates high levels of breeding site fidelity, while nest and chick census data in North Carolina, Maryland and Virginia indicates a gradual decrease in recruitment since the 1980s followed by a recent increase during the early 21^st ^Century [41]. Little is known about demographic patterns outside of these states and the species is classified with moderate concern in the North American waterbird conservation plan [42].

Here, we skimmed the genome through screening of 39 loci to identify fast-evolving nuclear markers that differentiate between closely related tern species. We then investigated (1) the phylogenetic origin and divergence of Sternidae, and (2) presence of nuclear polymorphism and genetic structure in different breeding populations of Royal Terns along the East Coast of the United States.

## Results

### Variation in amplification and intron length across species and loci

Amplification varied among loci and species but no correlation was observed between amplification success and chromosomal position or length of locus. Sixty-four percent of the markers that were tested amplified in at least one species (Table 1). Of these, some loci did not amplify consistently across samples, showed non-specific priming or did not sequence well. In total, 16 loci were used for further analyses (bold-faced in Table 1), including two mitochondrial markers and 14 intronic loci that vary in length from 236-844 bp. Length of each of the intronic loci did not vary significantly across the screened species. Comparison to published data for other neognathous birds for each of the 14 nuclear loci showed conserved length across neognathous birds in seven loci, increased length in terns in three loci, increased length in Neoaves in two markers and reduced length in terns at one locus. The 11^th ^intron of GAPDH showed significant length variation (270-428 bp) across several neognathous orders with terns displaying above average length: 380 bp, with an average of 340 bp per order. Three markers contained CR1 retroelement insertions in the Royal Tern. Locus 17483 contained a 303 bp insertion of the CR1-E family that explains the length discrepancy between tern and chicken/flycatcher. Locus 3862 contained a 134 bp insertion of the CR1-Y4 family, and Locus 26187 contained a 503 bp insertion of the CR1-Y2_Aves family. This marker was recently amplified in a population genetic study of meliphagid honey eaters [43] and corresponding sequences from this study contain a possible homologous insertion.

**Table 1:** Genetic loci screened in this study

Name	Chromosome	Function	Source
**ACL (16)**	27	ATP citrate lyase	[44]

AK1 (4-5)	17	Adenylate kinase 1	[44]

ALAS1 (8)	12	5-aminolevulinate synthase	[45]

AXIN (7)	14	Axin	[44]

BFIB* (7)	4	Beta-fibrinogen	[46]

BZW1 (6)	7	Basic Leucine Zipper Gene 1	This study

Brahma (15)	Z	Brahma/SMARCA-2	[45]

CEPU* (1)	24	CEPU/Neurotrimin	[44]

COI	MT	Cytochrome Oxidase I	[47]

**CRMIL (14)**	1	V-raf murine sarcoma viral oncogene	[44]

ENOL* (8)	21	Alpha-enolase	[48]

**G3PDH (11)**	1	Glyceraldehyde-3-phosphodehydrogenase	[49]

**GAPDH (4)**	1	Glyceraldehyde-3-phosphodehydrogenase	[50]

**Lamin (3)**	28	Lamin-A	[48]

**Control Region**	MT	D-loop of mitochondrial control region	This study, [11,51]

MPP (4)	4	Myelin proteolipid protein	[45]

MYO* (2)	1	Myoglobin	[52]

**ODC (6-7)**	10	Ornithine decarboxylase	[53]

P40 (5)	2	Laminin receptor precursor P40	[44]

R35*	9	G-protein coupled receptor R35, exon1	This study

**RGS4 (3)**	8	Regulator G-protein signaling 4	[44]

**VIM (7)**	2	Vimentin	[44]

**16S**	MT	16S rRNA (L2724-H3292)	[54]

3399	9	Pleckstrin homology domain-containing B2	[4]

**3862**	14	WD repeat protein 24	[4]

12884	1	Dihydrolipoamide dehydrogenase	[4]

13336*	10	Eukaryotic translation initiation factor 3-1α	[4]

**16264**	3	Postsynaptic protein CRIPT	[4]

**17483**	4	High mobility group protein B2	[4]

20352	2	Unknown	[4]

21277*	2	Unknown	[4]

**21281**	5	Vacuolar H+ ATPase E1	[4]

21571	1	Unknown	[4]

24813	1	Unknown	[4]

**25149**	4	Carboxypeptidase Z precursor	[4]

25442*	6	Ubiquitin-conjugating enzyme E2, J1	[4]

**26187**	1	ATPase, lysosomal accessory protein 2	[4]

27270	1	Phosphoglycerate dehydrogenase-like 1	[4]

27331*	1	Unknown	[4]

Each locus is shown with intron number in parentheses, chromosome location, gene function and source. Loci in bold are used in this paper for phylogenetic and/or population genetic analyses. Loci with an asterisk did not amplify or sequence consistently across species or showed non-specific priming problems

### Phylogeny of major groups of terns

**Figure 1:**
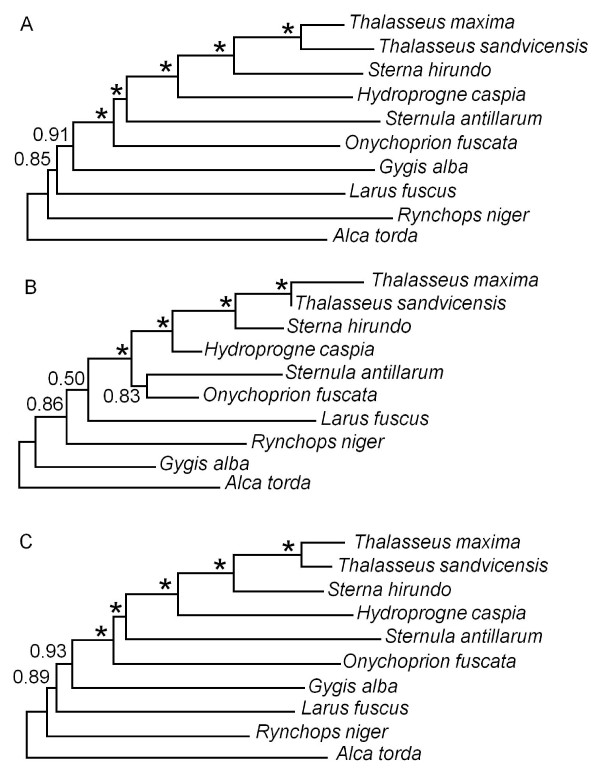
**Bayesian phylogeny of the major groups of terns and close relatives**. Bayesian posterior values are shown above the nodes, with asterisks denoting nodes with full support. **(1a) **analysis of the combined mitochondrial dataset (4616 bp: ND2, 12S+16S rRNA, Cytochrome b, Cytochrome Oxidase I); **(1b) **analysis of the combined nuclear data set (7026 bp: RAG-1 exon 1, Beta-fibrinogen intron 7, GAPDH intron 4 and 11, MYO intron 2, Lamin intron 3, ACL intron 16, and VIM intron 7); **(1c) **analysis of the full dataset containing mitochondrial and nuclear loci (11642 bp).

After addition of two mitochondrial markers (16S rRNA, COI) to previously published data, phylogenetic analysis continued to show support for the traditional relationships among terns and allies. Bayesian analyses of the combined mitochondrial data (4616 bp) showed a tern-gull grouping, monophyly of all terns and *Gygis *and *Onychoprion *as the oldest branches among terns (Figure 1a). Most of the nodes had full Bayesian and Maximum Likelihood bootstrap support. However, two nodes remain with near-significant support, those designating tern monophyly (BP = 0.91) and a tern-gull grouping (BP = 0.85). Analysis of a combination of protein-coding genes versus ribosomal genes indicated a conflict at the base of terns, with ribosomal genes excluding *Gygis *from a tern-gull group. This conflict remained when accounting for covarying sites in the rRNA and has been reported in other avian lineages [55].

Phylogenetic analyses of the nuclear loci included eight independent data sets comprised of both exonic (RAG1) and intronic (n = 7) loci data sets, four of which are new data sets in this context. Bayesian analysis of the combined eight loci (7026 bp) yielded a different yet-well supported phylogeny (Figure 1b) compared to the mitochondrial tree (Figure 1a). *Gygis*, the Angel Tern, was again the most basal tern but fell outside a grouping of other terns/gull/skimmer (BP = 0.86). In this tree, *Onychoprion *grouped with *Sternula *albeit without full Bayesian support (BP = 0.83). These results were not affected by excluding markers with missing data. None of the individual loci provided statistical support for the basal nodes, even though 5/8 loci suggested that *Gygis *should be excluded from a grouping of terns, gull and skimmer. The shortest locus, Lamin-A, instead supported a tern-*Gygis *grouping, like the mitochondrial protein-coding genes.

When all data were combined, a nearly fully supported Bayesian phylogeny was produced (Figure 1c) that was identical to the traditional/mitochondrial phylogeny. This phylogeny showed slightly increased support for the nodes in the mitochondrial phylogeny but still lacked full support.

### Variation among royal terns in twelve loci

Twelve loci consistently amplified across a number of Royal Terns, thus allowing investigation of polymorphic status and associated genetic structure for these loci. The amplicons derived from 11 nuclear intronic loci (Table 2) and the mitochondrial Control Region. The mitochondrial Control Region contained 11 variable sites and variation toward the end of the gene in the number of repeats of a short stretch of nucleotides. Out of these 11 variable sites, nine separated a previously published [11] sequence of a South American Royal Tern from the North American samples. The remaining two variable sites did not show fixed differences between the populations on the East Coast. Greater variation was found for the number of repeats of a microsatellite towards the 3'end of the Control Region. DNA sequences from Fisherman Island Terns displayed either six (n = 2), seven (n = 2) or 12 (n = 1) repeats, while sequences from Ferry Slip Island Terns displayed six (n = 2), seven (n = 1) or 21 (n = 1) repeats. Thus, with the exception of two longer repeats, microsatellite variation was greater within than between populations.

**Table 2:** Polymorphism information for 11 loci tested in Royal Tern

Gene	Length (bp)	Polymorphism (Royal Tern)	Polymorphism (other)	Polymorphism % (Royal Tern)	Reference taxon
**3862**	742	1/3	15/4	0.13	*Ficedula^a^*

**16264**	684	1/3	11/10	0.15	*Ficedula^a^*

**17483**	806	1/10	7/4	0.12	*Ficedula^a^*

**21281**	639	0/4	10/10	0	*Ficedula^a^*

**25149**	844	0/5	14/9	0	*Ficedula^a^*

**26187**	868	3/6	16/10	0.35	*Ficedula^a^*

**ACL**	458	2/10	3/38	0.44	*Larus^b^*

**CRMIL**	630	2/5	7/18	0.32	*Larus^b^*

**G3PDH**	380	1/5	3/50	0.26	*Sternula^c^*

**Lamin**	236	0/4	10/32	0	*Pterodroma^d^*

**RGS4**	745	6/6	6/17	0.81	*Larus^c^*

Polymorphism is shown as number of variable sites/number of sampled terns and% of variable sites, compared to values reported from the phylogenetically closest available lineage in literature (^a^[4]; ^b^[44]; ^c^[56]; ^d^[57]).

**Figure 2:**
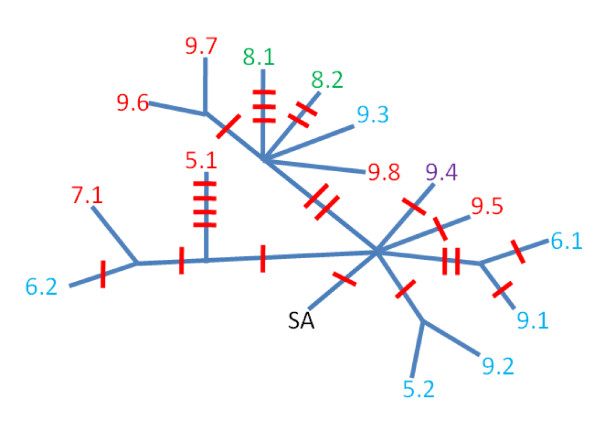
**Genotype network of the various Royal Terns used in the population genetic analysis**. Royal Terns from Ferry Slip Island are red (extraction) numbers, Fisherman Island terns are blue, Texas terns are green, the Bigfoot Island Tern is purple, and the GenBank tern from French Guiana is shown as SA in black. Red dashes represent genes containing at least one polymorphic site.

Eight of the eleven nuclear loci also identified polymorphism within the sampled East Coast Royal Terns, which ranged from one to six variable sites. The presence of polymorphism at this geographic scale for Royal Terns agrees with similar values from studies in Collared Flycatcher, Australian Honeyeater and Kelp Gull that sampled a comparable or larger geographic range. This result is particularly true for RGS4, CRMIL and 26187. However, other loci, e.g. Lamin, 21281 and 25149, do not follow this correlation across distantly related species (Table 2). With the exception of three loci, the terns from the East Coast did not show fixed differences from the two sampled Texan terns. The majority of polymorphisms indicated variation within NC and VA populations (Table 3), in agreement with the mitochondrial findings. A minimal spanning network was created to show the effect of accumulating differences across loci and individuals (Figure 2), indicating one sampled tern (Ferry Slip Island, NC) that differed genetically from others at several (n = 5) loci.

**Table 3:** Genotypes/haplotypes as shown by various loci in Royal Terns

Gene	1	2	3	4	5	6	7	8	Unresolved
**Mt-CR**	TX1/FI1/FS3	FS1/FI1	FI1	FS1	GB	FI1	FI1	FS1	FI1

**RGS4**	TX1	FS1	FI1	TX1	FI1	FI1	-	-	-

**CRMIL**	TX1/FS1	FS2	BF1	-	-	-	-	-	-

**g3pdh**	TX1/FS2	TX1	FS1	-	-	-	-	-	-

**ACL**	BF1/FS4/FI2	FS1	FI2	-	-	-	-	-	-

**3862**	TX1	FS1	-	-	-	-	-	-	FI1

**16264**	TX2	FS1	-	-	-	-	-	-	-

**17483**	BF1/FS5/FI2	FI1/FS1	-	-	-	-	-	-	-

**26187**	TX1/BF1/FS3	TX1	-	-	-	-	-	-	-

**Lamin**	FS2/FI3	-	-	-	-	-	-	-	-

**25149**	FS3/FI1/BF1	-	-	-	-	-	-	-	-

**21281**	TX1/FS1/FI2	-	-	-	-	-	-	-	-

Sample localities are shown in abbreviation jointly with sample number. *FS *ferry slip island, *FI *fisherman island, *TX *Texas, *BF *Bigfoot island, *GB *GenBank sequence from French Guiana. Unresolved refers to ambiguous sequence information, which was not taken into account in the polymorphism analysis.

## Discussion

In conjunction with mitochondrial and microsatellite length variation, mutational variation may accumulate in nuclear introns during the evolutionary history of avian populations. Considering the genetic challenges that past tern studies have faced, the screening of introns may thus provide a rich tool for investigation of genetic structure and diversity. Such intronic sequence data may serve a dual role in also providing phylogenetic resolution among closely related species. Through skimming of the Royal Tern's genome via screening of polymorphic introns located on various chromosomes, this study was successful in identifying novel markers for tern population genetic investigation. These new data provide support, albeit preliminary, for genetic connectivity among Royal Terns from Virginia to southern North Carolina. Phylogenetic insight is also obtained from several new introns on the evolutionary history of terns, particularly the linear sequence of the primitive lineages of brown-winged, little and angel terns.

Particularly promising for avian population genetic studies was the successful amplification of > 50% of loci that were previously tested in a variety of birds, but not in terns or other shorebirds. Although many of the primers target conserved flanking exons and were designed with sequence comparison between galloanserine and neoavian species in mind, the suitability of these loci and their primers for non-model neognathous birds, such as terns, is no guarantee. Several reasons can limit the efficacy of primers with "broad" taxonomic utility. These reasons include genome-wide mutation rate differences among major bird lineages, e.g. increased mutation rates in songbirds and decreased mutation rates in aquatic birds [8,9,17], lineage-specific mutation in the primer region, and extensive intron length variation due to retroelement insertion. Because of consistent amplification success for the loci described here, future population genetic study on terns should consider including the polymorphic loci highlighted here. The increased statistical power of a multi-locus approach in population genetics and phylogeography has been promoted before (e.g. [1]), and is indeed attainable for non-model birds as suggested by [7] and confirmed here. The slow rate of the mitochondrial Control Region was confirmed for Royal Terns, but the presence of a highly polymorphic tandem repeat at the 3'end of the Control Region makes this marker nonetheless useful at the population scale. Interestingly, the presence of this repeat overlaps with a heteroplasmic area in gull species [11] but did not show intra-individual variation in any of the Royal Terns studied here. The value of these repeats for genetic studies of different tern species should be assessed on a species by species basis.

At first glance, the presence of shared genotypes shown in several loci from Virginia to the Southern end of North Carolina does not agree with the finding of extensive natal and breeding site fidelity in Royal Terns observed in North Carolina. However, these findings are in line with studies of other tern species. Both Sooty and Angel Terns show extensive natal philopatry but lack genetic structure across a large geographic range [32,35]. Secondly, due to the preference for vegetation-sparse habitats during the breeding season, plant succession will lead to colony abandonment in Royal Terns [41]. Thus Royal Terns, like other seabirds, may show strong nesting fidelity over several years with occasional interruption. Such events would increase gene flow among nearby colonies, and prevent development of genetic distinctness over evolutionary time. Alternatively, it is possible that shallow genetic structure does exist but was not discovered with our current sampling. Both alternatives would benefit from additional data from other parts of the Royal Tern's range.

Despite addition of more data, phylogenetic analyses confirm a conflict at the base of Sterninae. It remains unclear whether the species tree from the longer coding portion of the mitochondrial genomes or that from most individual nuclear loci and the mt rRNA is correct. Uncertainty about the phylogenetic relationships of the genus *Gygis *and *Onychoprion *was apparent from comparison of previous mitochondrial-based and nuclear-based phylogenetic studies. Yet, these studies differed in species sampling and were limited to one or few nuclear loci in combination with mitochondrial data. Here, available partial mitochondrion data were combined with an additional mitochondrial region. Results confirm the phylogenetic consistency across mitochondrial protein-coding genes in supporting the Bridge et al. [14] tree. However, when combined, the 12S and 16S rRNA genes, appear in more agreement with previous and new nuclear sequence results, particularly those based on the large RAG1 exon [28]. Nonetheless, the results from analysis of all data suggests that the mitochondrial protein-coding signal is stronger than the nuclear or rRNA signal. Considering that the conflict over the true nature of the Angel Tern resides at or near the juncture of the initial diversification of terns, gulls and skimmers, which likely occurred over a short period of time [29], future phylogenetic resolution would benefit from additional genetic data from more loci and relevant taxa (e.g. *Anous, Procelsterna*), including sampling more individuals per species to ascertain individual gene tree coalescence patterns.

## Conclusions

Choosing appropriate molecular markers in ecology and evolution can be a daunting task when working with non-model species. Terns and their allies offer additional genomic and technical challenges. We have skimmed various intronic regions across the tern, skimmer and gull nuclear genome, and here have documented the value of intron-based loci to both phylogenetic and population study of terns. We expect that these markers will comprise a valuable tool for future genetic investigation into the evolution, ecology and conservation of terns, and a viable alternative to mitochondrial and microsatellite techniques. We also recommend screening of additional introns to increase statistical and phylogenetic power in avian genetics, through standard PCR-based genome skimming or next-generation sequencing methods.

## Methods

Samples of tern species (Additional file 2: Table S2) were selected to maximize generic representation of the 45 known species of terns, the Lesser Black-Backed Gull (*Larus fuscus*) and Black Skimmer (*Rynchops niger*) were chosen as representative of potential sister group lineage (gulls and skimmers), and the Razorbill (*Alca torda*) was used as the outgroup. The sampled tern species included two representatives of the genus *Thalasseus*, the Royal Tern (*T*. *maxima*) and Sandwich Tern (*T*. *sandvicensis*); one *Hydroprogne*, the Caspian Tern (*H. caspia*); one *Sternula*, the Least Tern (*S. antillarum*); one *Onychoprion*, the Sooty Tern (*O*. *fuscata*), one *Sterna*, The Common Tern (*S*. *hirundo*), and one *Gygis*, the Angel Tern (*G*. *alba*). Representative samples from the genus *Phaetusa*, *Anous *or *Procelsterna*, relevant to investigating the linear sequence at the base of Sterninae [14], were not available at the time of study. Previously published tern sequences were downloaded from Genbank (NCBI 2010) and included in our phylogenetic analysis when most representative taxa were available. These sequences derived from both mitochondrial (ND2, 12S rRNA, 16S rRNA, Cytochrome b [CYTB], Cytochrome oxidase 1 [COI], Control Region) and nuclear (RAG-1, beta-fibrinogen [BFIB] intron 7, GAPDH intron 4, and Myoglobin [MYO] intron 2) loci.

Fifteen Royal Terns were included in population genetic analysis and derived from populations along the East Coast of North America and Texas (Additional file 2: Table S2). The samples included 6 Royal Terns from Ferry Slip island at the mouth of the Cape Fear River near Southport, NC, 6 Royal Terns from Fisherman's Island National Wildlife Refuge, VA, 1 Royal Tern from Bigfoot Island near Ocracoke Island, NC, and 2 Royal Terns from Texas that served as comparison to the East Coast samples. The Texas terns were originally collected at the Texas Coast near Houston and near the Mexico border. The East Coast tern samples included blood material previously collected by SDE and students (T. Maness, M. Meadows) in banding expeditions. All research was in accordance with UNCW's institutional guidelines for animal care; blood samples were obtained using Institutional Animal Care Use Committee protocol (UNCW: 2005-002).

DNA extraction process consisted of cutting tissue samples with a razor blade, and processing about 20 mg of cut tissue with the Qiagen DNeasy tissue extraction protocol. Ten ul of blood samples were premixed with PBS and processed using the Qiagen DNeasy blood extraction protocol. To quantify the amount of DNA, samples were analyzed on a NanoDrop 2000 machine. Primers were chosen from avian molecular literature resources or designed specifically for this study with the IDTDNA Oligo-Analyzer http://www.idtdna.com/analyzer/applications/oligoanalyzer/. Loci were selected that showed polymorphism in other avian species, most of which derived from *Ficedula *flycatchers [7,45], but also included previously tested markers in close relatives of terns, particularly the gulls [44] and murrelets [48]. Due to the large number of available primers from the *Ficedula *genome, a subset of loci (n = 16) were selected that showed polymorphism to varying degrees in *Ficedula albicollis *and that encompassed a range of intron length (200-900 bp). The partial Mitochondrial Control Region was sequenced using two available primers (L-438: [51], H-1248: [11]), and newly designed primers (Tern-L1000: CATCATCTTTGTTACACGTCAAC, Tern-H1000: GTTGACGTGTAACAAAGATGATG). For two additional loci, primers were also newly designed: Basic Leucine Zipper (BZW1-F: GGTATTCTGCTWGCCAATGGGAC, BZW1-R: GTTTGTTGGCTGGGAAGAGTTCC) and R35 (R35F: GTGCCAGTGATTATTTTCATGCTC, R35R: GGARAKGCTYCARGGTYTTATCC). The total list of screened loci is summarized in Table 1, and PCR conditions are given for loci used in further analysis in Additional file 3: Table S3.

For each Polymerase Chain Reaction (PCR), 2.5 μL DNA sample (10 μM) was mixed with 31.0 μL distilled water and 16.5 μL of a mixture containing 5 μL PCR Buffer (10×), 5 μL MgCl_2 _(25 mM), 1 μL dNTPs (10 mM each), 2.6 μl forward primer (5 μM), 2.6 μL reverse primer (5 μM) and 0.2 μL Multi Taq DNA Polymerase at 5 U/μL. PCR conditions included a 3 min warm-up period at 95°C, followed by 40 cycles of 30 s at 95°C, 30 s at 50°C and 30 s at 72°C. When amplification failed under these conditions, PCR conditions were modified by increasing annealing and extension time to 45 s and/or increasing the annealing temperature to 55°C. PCR products were inspected after gel electrophoresis on a 2% Agarose gel and successful products cleaned using modified ExoSap conditions (42 ul of PCR products were mixed with 1.45 μL Exonuclease I (5 U/μL), 2.85 μL Shrimp Alkaline Phosphatase (1 U/μL), and 2.85 μL denucleated water, heated at 37°C for 30 min, and 80°C for 15 min. DNA sequencing was outsourced to Macrogen Inc. Korea. DNA sequences were cleaned and aligned in Sequencher v5.0 (Genecodes Corp.).

Phylogenetic analysis was performed using the composite Maximum Likelihood method, bootstrapping and a pairwise deletion option in MEGA v4.0 [58] and by performing three Bayesian MCMC runs of 10 million iterations with the GTR+G+I, uncorrelated lognormal clock and Yule speciation models in BEAST v1.4.8 with default priors. Percent burnin, effective sample size and convergence among runs were assessed with Tracer v.1.4.1 and topologies with posterior probability constructed after removal of burnin using TreeAnnotator v.1.4.8 and FigTree v1.2.2. Locus-specific polymorphic sites were documented in Sequencher v5.0 from consensus sequences obtained from the forward and reverse strands, and using IUPAC codes for heterozygous sites, all of which were of the transitional type (R and Y). Corresponding Genbank accession numbers for new DNA sequences are Genbank: X001-Y100 (will be updated). A nexus file containing new molecular sequences is available as a link to this paper (Additional file 4).

## Abbreviations

BP: Bayesian posterior values; bp: Base pairs; CR1: Chicken repeat 1; U, Unit.

## Competing interests

The authors declare that they have no competing interests.

## Authors' contributions

DGJ carried out the molecular genetic studies, participated in the sequence alignment and data analysis, and drafted the manuscript. SDE participated in the design of the study, collected field samples and helped to draft the manuscript. MVT conceived of the study, participated in the design of the study, participated in analysis of the data, helped to draft the manuscript and provided funding for the project. All authors read and approved the final manuscript.

## Authors' information

DGJ is a recently graduated student who performed the work described in this paper as part of his undergraduate honors project. He is interested in applied animal and human health-related sciences. SDE is a full professor in the Department of Biology and Marine Biology at UNCW and is broadly interested in bird and mammal (paleo)-ecology. MVT is an assistant professor at the Department of Biology and Marine Biology, UNCW, where he studies patterns of bird and mammal biodiversity through time with a combination of genetics, fossils and ecology.

## Supplementary Material

Additional file 1 **Table S1**. Summary of published tern population genetic studies and their main findings. Name, reference, data set and main results are given for genetic studies on seven tern species [59]. Click here for file

Additional file 2 **Table S2**. Sample information for terns and allies used in this study. Extraction number, name, museum catalogue number and collection locality are given for each individual sampled. NCSM = North Carolina Natural Sciences Museum; BPBM = Bernice P. Bishop Museum; MSB = Museum of Southwestern Biology; FS = Ferry Slip Island (UNCW), FI-Fisherman Island (UNCW), BF = Bigfoot Island. Click here for file

Additional file 3 **Table S3**. PCR information for primers used in population genetic or phylogenetic analysis. Primer names are given for loci used in analysis (boldfaced and asterisked loci in Table 1), along with representative annealing temperatures used in successful PCR (all run with 40 cycles), forward and reverse primer sequences, and successfully amplified species. See Table S2 for source of extraction name. Click here for file

Additional file 4 **Terngenelist.nex**. Combined sequence data in nexus format used in phylogenetic reconstruction. Click here for file
