# Supplementary material for: Genome skimming identifies polymorphism in tern populations and species
Source: BMC Res Notes. 2012 Feb 14;5:94. doi: 10.1186/1756-0500-5-94 (PMC3292991; doi:10.1186/1756-0500-5-94)
Supplement: Additional file 1 — Table S1. Summary of published tern population genetic studies and their main findings. Name, reference, data set and main results are given for genetic studies on seven tern species [59]. [file 1756-0500-5-94-S1.DOCX]

**Table S1.** Summary of published tern population genetic studies and their main findings.

| Common Name | Papers | Loci | Results |
| --- | --- | --- | --- |
| Angel Tern (G. alba) | Yeung et al. 2009 | mt-ND2/CYTB | Extensive gene flow among all populations; merging of 2 species and 2/4 subspecies |
| Least Tern (S. antillarum) | Whittier et al. 2006 Draheim 2010 | microsatellites mt-CR/CYTB nuc-G3PDH | Homogeneity within populations; extensive gene flow between eastern and western US populations |
| Roseate Tern (S. dougalli) | Lashko 2004 Szczys et al. 2005 | microsatellites mt-ND2/ND6 | Significant variation between Indopacific and Atlantic population; homogeneity within populations; recent expansion in both oceans; merging of 3/4 subspecies |
| Sooty Tern (O. fuscata) | Avise et al. 2000 Peck, Congdon 2004 | mt-CR RFLP | Low but significant variation between Indopacific and Atlantic population; homogeneity within populations |
| S. American Tern (*S. hirundinacea*) | Faria et al. 2010 | microsatellites mt-ND2/CYTB | Low but significant variation between Brazilian and Patagonian population; homogeneity within populations |
| Common Tern (S. hirundo) | Burson 1990 Sruoga et al. 2006 | microsatellites isoelectric protein focusing | Extensive gene flow between adjacent states (MN, WI); low but significant variation among breeding colonies in Lithuania |
| Sandwich Tern (T. sandvicensis) | Efe et al. 2009 | microsatellites mt-ND2/ND6/CYTB nuc-BFIB/MYO | Old World and New World terns are different species; low differentiation among North American and South American terns with complex genetic structure and gene flow |
