# Supplementary material for: Genome skimming identifies polymorphism in tern populations and species
Source: BMC Res Notes. 2012 Feb 14;5:94. doi: 10.1186/1756-0500-5-94 (PMC3292991; doi:10.1186/1756-0500-5-94)
Supplement: Additional file 2 — Table S2. Sample information for terns and allies used in this study. Extraction number, name, museum catalogue number and collection locality are given for each individual sampled. NCSM = North Carolina Natural Sciences Museum; BPBM = Bernice P. Bishop Museum; MSB = Museum of Southwestern Biology; FS = Ferry Slip Island (UNCW), FI-Fisherman Island (UNCW), BF = Bigfoot Island. [file 1756-0500-5-94-S2.DOCX]

**Table S2.** Sample information for terns and allies used in this study.

| Extraction No. | Species | Catalogue No. | Location |
| --- | --- | --- | --- |
| 1.1 | *Thalasseus maxima* | NCSM20951 | Holden Beach, Brunswick Co., NC |
| 1.2/2.2/3/3 | *Hydroprogne caspia* | NCSM20702 | Morehead City, Carteret Co., NC |
| 1.3/2.3/4.2 | *Sternula antillarum* | NCSM20827 | New Bern, Craven Co., NC |
| 1.4/2.4/4.3 | *Onychoprion fuscata* | NCSM20813 | Jacksonville, Onslow Co., NC |
| 1.5/2.5/4.4 | *Thalasseus sandvicensis* | NCSM20805 | Hammocks Beach, Carteret Co., NC |
| 1.6/2.6/4.5 | *Larus fuscus* | NCSM1001 | Dare Co., NC |
| 1.7/7.3 | *Rynchops niger* | NCSM23076 | Dare Co., NC |
| 2.1/4.1 | *Thalasseus maxima* | NCSM22697 | Unknown |
| 3.1 | *Gygis alba* | BPBM184991 | Maui, HI |
| 3.2 | *Sterna hirundo* | NCSM23437 | Unknown |
| 5.1 | *Thalasseus maxima* | FS69 | Ferry Slip Island, Cape Fear River, NC |
| 5.2 | *Thalasseus maxima* | FI37 | Fisherman's Island NWR, VA |
| 6.1 | *Thalasseus maxima* | FI125 | Fisherman's Island NWR, VA |
| 6.2 | *Thalasseus maxima* | FI141 | Fisherman's Island NWR, VA |
| 7.1 | *Thalasseus maxima* | FS80 | Ferry Slip Island, Cape Fear River, NC |
| 7.2/9.8 | *Thalasseus maxima* | FS101 | Ferry Slip Island, Cape Fear River, NC |
| 8.1 | *Thalasseus maxima* | MSB24233 | High Island, Chambers Co., TX |
| 8.2 | *Thalasseus maxima* | MSB29439 | South Padre Island, Cameron Co., TX |
| 9.1 | *Thalasseus maxima* | FI85 | Fisherman's Island NWR, VA |
| 9.2 | *Thalasseus maxima* | FI100 | Fisherman's Island NWR, VA |
| 9.3 | *Thalasseus maxima* | FI130 | Fisherman's Island NWR, VA |
| 9.4 | *Thalasseus maxima* | BF79 | Bigfoot Island, near Ocracoke, NC |
| 9.5 | *Thalasseus maxima* | FS22 | Ferry Slip Island, Cape Fear River, NC |
| 9.6 | *Thalasseus maxima* | FS34 | Ferry Slip Island, Cape Fear River, NC |
| 9.7 | *Thalasseus maxima* | FS76 | Ferry Slip Island, Cape Fear River, NC |
