# Supplementary material for: Genome skimming identifies polymorphism in tern populations and species
Source: BMC Res Notes. 2012 Feb 14;5:94. doi: 10.1186/1756-0500-5-94 (PMC3292991; doi:10.1186/1756-0500-5-94)
Supplement: Additional file 4 — Terngenelist.nex. Combined sequence data in nexus format used in phylogenetic reconstruction. [file 1756-0500-5-94-S4.DOC]

**Table S3. - PCR information for primers used in population genetic or phylogenetic analysis.**

| **name** | **Ta** | | **Primers** | **Species PCRed/sequenced** |
| --- | --- | --- | --- | --- |
| 13336 | 50 | CAGTTAGCAGACAAACTACG  GGTTCATGGCATCTATTCC | | Royal Tern |
| 16264 | 50 | GCATTGACCTCAAAGAAGGC  TTTATAGGCACATCCTTGAC | | Royal Tern, FS69, 24233, 29439 |
| 16S rRNA | 55 | CGAGCYKGGTGATAGCTGG  TGATTRYGCTACCTTYGCACGG | | All species except Angel Tern and Common Tern; Sandwich Tern did not sequence well |
| 17483 | 52 | GAAATGTGGTCTGAACAGTC  TTGCTCTTGGCACGATATGC | | Royal Tern, FS69, FI125, FI141, FS80, FI85, BF79, FS22, FS34, FS76, FS101 |
| 21277 | 55 | TACATAGCCGATATGCTAAC  AGGTTTCCACGCTTCTTGTC | | Royal Tern |
| 21281 | 50 | GACCAAGACAACTTCCTGCC  GTAGATTTCGACACCTCCAG | | Royal Tern, FS69, FI125, FI141, 29439 |
| 25149 | 50 | TGGCCCTACTTCTTAGACTG  CAATGTGCTCACCATTTGGG | | Royal Tern and Sooty Tern, FS69, FI125, BF79, FS34, FS76 |
| 25442 | 52 | CCTGAATATCCCATGAAACC  GCTGCCATGTTTCAGGATG | | Royal Tern |
| 26187 | 50 | GGTGGGAATGCAGTAGTAGA  CATGTTCCAGAGGTTGTAGG | | Royal Tern, FS69, FI125, FI141, FS80, 24233, 29439, BF79, FS76 |
| 27331 | 55 | CCTAGCTAAATATGTTCTGGC  TAGGCTTCCTGATGATGGCT | | Royal Tern |
| 3862 | 55 | CCCTCCGACTTCTTCAACCC  CACCTCGTTGGAGATGTTCC | | Royal Tern and Sooty Tern, FS69, FI125, 24233 |
| ACL (16) | 55 | GCTCTGCTTATGACAGCACT  CAGCAATAATGGCAATGGTG | | Royal Tern, Sooty Tern, Gull, Angel Tern, and Common Tern, FS69, FI125, FI85, FI100, FI130, BF79, FS22, FS34, FS76, FS101 |
| BFIB (7) | 52 | GGAGAAAACAGGACAATGACAATTCAC  TCCCCAGTAGTATCTGCCATTAGGGTTT | | None sequenced |
| CEPU (1) | 55 | CGAGTCAAAGTCACCGTCAA  CTCTTCGCATCCGAGATGTA | | Royal Tern |
| Control Region | 55 | TCACGTGAAATCAGCAACCC  GTTGACGTGTAACAAAGATGATG;  CATCATCTTTGTTACACGTCAAC  CATCTTCAGTGCCATGCTTT | | Least Tern, Gull, Common Tern, FS69, FI37, FI125, FI141, FS80, 29439, FI85, FI100, FI130, FS22, FS34, FS101 |
| CRMIL (14) | 50 | TCAATCATCCACAGAGACC  TGATGAGATCCACTCCATCG | | Royal Tern, FS69 |
| ENOL (8) | 55 | GACTTCAAATCYCCYGATGAYCCCAG  CCAGTCRTCYTGGTCAAADGGRTCYTC | | Royal Tern, Sooty Tern, and Skimmer, did not sequence |
| G3PDH (11) | 52 | GGCATTGCACTGARYGAYCATTT  ARRTCCACAACACGGTTGCTGTA | | All species except Caspian Tern and Angel Tern; FS69, 24233, 29439, FS76, FS101 |
| GAPDH (4) | 55 | TCTCTGGCAAAGTCCAAGTG  TCATGGTTGACACCCATC | | Angel Tern, others from Genbank |
| Lamin (3) | 50 | CCAAGAAGCAGCTGCAGGATGAGATGC  CTGCCGCCCGTTGTCGATCTCCACCAG | | All species, FS69, FI37, FI125, FI141 |
| MYO (2) | 50 | GCCACCAAGCACAAGATCCC  CCAGACTAAGAAATAGGTTGC | | None sequenced |
| ODC (6-7) | 50 | CTCCAAAGCAGTTTGTCGTCTCAGTGT  TCTTCAGAGCCAGGGAAGCCACCACC | | Gull, others from Genbank |
| R35 | 50 | GTGCCAGTGATTATTTTCATGCTC  GGARAKGCTYCARGGTYTTATCC | | Royal Tern |
| RGS4 (3) | 52 | TCGCTGGAAAACTTGATCC  GTAGTCCTCACAACTGACC | | Royal Tern, FS69, FI37, FI125, FI141, 24233, 29439 |
| VIM (7) | 52 | TGCTTCTTTGAACCTGAGAG  GTGTCCTCTTCGAGTGAGTG | | Royal Tern, Sooty Tern, Angel Tern, and Common Tern, FI 125 |
